# Letter from Dr. C. M. Rosser

**Published:** 1890-02

**Authors:** C. M. Rosser

**Affiliations:** Dallas, Texas


### LETTER FROM DR. ROSSER.

DALLAS, TEXAS, January 27, 1890.

*Editor Daniel's Texas Medical Journal:*

Having severed my editorial connection with the Texas Courier-Record of Medicine, I am of course deprived of the very valuable exchanges that it has been my good fortune to enjoy, and not wishing to be interrupted in the reading of your JOURNAL, I herewith hand you $2 and advise you to number me among your subscribers and supporters.

I have appreciated your friendly courtesies during the brief period in which I had the honor to be an editorial contemporary, and although I have retired from the journalistic field, I have still the same regard and enthusiasm toward the profession of Texas, and for her literature and institutions. I have daily more reason to be proud that I am in some manner identified with them.

Recently I had the pleasure of being entertained by my friend Dr. Paine, in Galveston, at which time I took occasion to visit the Texas Medical College and Hospital. I freely confess that we have there a nucleus of which I had no intelligent idea.

The school is equipped very well in a general way, and the department of Dr. Dock has bsen furnished with facilities for teaching pathology and microscopy, of which the majority of older schools can not boast.

The John Sealy Hospital which is a magnificent structure, costing about $70,000, supplies ample clinical material. Of the faculty, nothing of their well earned character need be said, for they are our friends and neighbors, and we know them. And now Texas on account of her medical institution, her medical press, her medical associations and her medical profession may well be congratulated. Truly yours,

[C. M. ROSSER.

